# MUSCLE GROUP SPECIFIC SKELETAL MUSCLE AGING: A FIVE YEAR LONGITUDINAL STUDY IN SEPTUAGENARIANS

**DOI:** 10.1093/geroni/igac059.2887

**Published:** 2022-12-20

**Authors:** Masatoshi Naruse, William Fountain, Alex Claiborne, Holmes Finch, Scott Trappe, Todd Trappe

**Affiliations:** Ball State University, Muncie, Indiana, United States; Ball State University, Muncie, Indiana, United States; Ball State University, Muncie, Indiana, United States; Ball State University, Muncie, Indiana, United States; Ball State University, Muncie, Indiana, United States; Ball State University, Muncie, Indiana, United States

## Abstract

Most of the scientific literature on human skeletal muscle aging has focused on the quadriceps femoris, with the remaining few hundred skeletal muscles receiving limited specific investigation. This longitudinal investigation compared changes in skeletal muscle size via computed tomography of the quadriceps (rectus femoris, vastus lateralis, vastus medialis, vastus intermedius), hamstrings (biceps femoris short and long heads, semitendinosus, semimembranosus), psoas, rectus abdominis, lateral abdominals (obliques and transversus abdominus), and paraspinal muscles (erector spinae and multifidi) of older individuals from the Health ABC study at baseline and 5.0±0.1 years later (n=469, 73±3y & 78±3y, 49% women, 33% black). Skeletal muscle size decreased (p < 0.05) in quadriceps (-3.3%), hamstrings (-5.9%), psoas (-0.4%), and rectus abdominus (-7.0%). The hamstrings and rectus abdominus atrophied approximately twice as much as the quadriceps (p < 0.05), while the quadriceps atrophied substantially more than the psoas (p < 0.05). The paraspinals (+4.3%) and lateral abdominals (+5.9%) hypertrophied (p < 0.05) to a similar degree (p>0.05) over the 5 years. These data suggest that skeletal muscle mass in older individuals changes in a muscle group specific fashion in the eighth decade, a critical time period in the aging process. A broader understanding of muscle group specific skeletal muscle aging is needed to better guide exercise programs and other interventions that mitigate decrements in physical function with aging.

